# *SND2*, a NAC transcription factor gene, regulates genes involved in secondary cell wall development in *Arabidopsis *fibres and increases fibre cell area in *Eucalyptus*

**DOI:** 10.1186/1471-2229-11-173

**Published:** 2011-12-01

**Authors:** Steven G Hussey, Eshchar Mizrachi, Antanas V Spokevicius, Gerd Bossinger, Dave K Berger, Alexander A Myburg

**Affiliations:** 1 Department of Genetics, Forestry and Agricultural Biotechnology Institute (FABI), University of Pretoria, Pretoria, 0002, South Africa; 2 Department of Forest and Ecosystem Science, The University of Melbourne, Melbourne, 3363, Australia; 3 Department of Plant Science, Forestry and Agricultural Biotechnology Institute (FABI), University of Pretoria, Pretoria, 0002, South Africa

## Abstract

**Background:**

NAC domain transcription factors initiate secondary cell wall biosynthesis in *Arabidopsis *fibres and vessels by activating numerous transcriptional regulators and biosynthetic genes. NAC family member *SND2 *is an indirect target of a principal regulator of fibre secondary cell wall formation, SND1. A previous study showed that overexpression of *SND2 *produced a fibre cell-specific increase in secondary cell wall thickness in *Arabidopsis *stems, and that the protein was able to transactivate the *cellulose synthase8 *(*CesA8*) promoter. However, the full repertoire of genes regulated by *SND2 *is unknown, and the effect of its overexpression on cell wall chemistry remains unexplored.

**Results:**

We overexpressed *SND2 *in *Arabidopsis *and analyzed homozygous lines with regards to stem chemistry, biomass and fibre secondary cell wall thickness. A line showing upregulation of *CesA8 *was selected for transcriptome-wide gene expression profiling. We found evidence for upregulation of biosynthetic genes associated with cellulose, xylan, mannan and lignin polymerization in this line, in agreement with significant co-expression of these genes with native *SND2 *transcripts according to public microarray repositories. Only minor alterations in cell wall chemistry were detected. Transcription factor *MYB103*, in addition to *SND1*, was upregulated in *SND2*-overexpressing plants, and we detected upregulation of genes encoding components of a signal transduction machinery recently proposed to initiate secondary cell wall formation. Several homozygous T4 and hemizygous T1 transgenic lines with pronounced *SND2 *overexpression levels revealed a negative impact on fibre wall deposition, which may be indirectly attributable to excessive overexpression rather than co-suppression. Conversely, overexpression of *SND2 *in *Eucalyptus *stems led to increased fibre cross-sectional cell area.

**Conclusions:**

This study supports a function for *SND2 *in the regulation of cellulose and hemicellulose biosynthetic genes in addition of those involved in lignin polymerization and signalling. SND2 seems to occupy a subordinate but central tier in the secondary cell wall transcriptional network. Our results reveal phenotypic differences in the effect of *SND2 *overexpression between woody and herbaceous stems and emphasize the importance of expression thresholds in transcription factor studies.

## Background

Plant fibres constitute a valuable renewable resource for pulp, paper and bioenergy production [1]. In angiosperms, the two principle sclerenchyma cell types that comprise secondary xylem are xylem vessels, which facilitate the transport of water, and xylary fibres, which provide mechanical strength and which make up the bulk of woody biomass [2]. Wood density and chemical composition, fibre and vessel length, diameter and wall thickness, and even the proportion of axial and radial parenchyma heavily influence pulp yield, digestibility and quality, although the relative importance of each varies from species to species [3,4].

During xylogenesis in angiosperms, fibres differentiate from the vascular cambium, elongate, and deposit a lignified secondary cell wall (SCW). SCW formation is associated with a distinct form of programmed cell death [5,6]. Much research has been devoted to the biosynthesis of SCW biopolymers, namely (in decreasing order of abundance) cellulose [7,8], hemicellulose [9] and lignin [10,11]. Complementing this, in the past six years much of the transcriptional network underlying SCW biosynthesis has been deciphered, mainly exploiting *Arabidopsis thaliana *and the *Zinnia elegans *mesophyll-to-tracheary element *in vitro *transdifferentiation system [12,13]. Genes involved in secondary xylem formation are regulated principally at the transcriptional level, accentuating the central significance of the SCW transcriptional network [14]. Manipulation of transcription factors (TFs) associated with the network presents the potential to enhance fibre properties through altering the regulation of a large number of biosynthetic genes.

Kubo *et al. *[15] first identified NAC domain TFs VASCULAR-RELATED NAC-DOMAIN7 (VND7) and VND6 as "master activators" of SCW formation in proto- and metaxylem vessels, respectively. It was later shown that VND6 and VND7 are functionally redundant, being sufficient for all vessel SCW formation [16,17]. In xylem fibres, a similar transcriptional master switch was identified. NAC family proteins SECONDARY WALL-ASSOCIATED NAC DOMAIN1 (SND1) and NAC SECONDARY WALL THICKENING PROMOTING FACTOR1 (NST1) redundantly activate *Arabidopsis *fibre (and, to some extent, silique valve) SCW formation [18-22]. In other cell types with secondary walls, such as the endothecium of anthers, NST1 was also found to activate SCW development, in this case redundantly with NST2 [23]. Together, these studies support a role for NAC TFs as principal activators of SCW formation in fibres and vessels, acting in distinct combinations in each case.

Several studies suggest that SND1, NST1, VND6 and VND7 activate a conserved, cascading transcriptional network featuring, but by no means limited to, various NAC, MYB and homeodomain TFs (reviewed in [13,24]). SND1, NST1, NST2, VND6 and VND7 regulate an overlapping set of targets [21,25], supported by the ability of NST2, VND6 and VND7 to complement the *snd1 nst1 *double mutant when ectopically expressed in fibre cells [26,27]. For this reason, they have been collectively referred to as secondary wall NACs (SWNs) [26]. Amongst the downstream targets of SWNs, *SND3 *and *MYB103 *are directly activated by SND1/NST1 and VND6/VND7 [21,25-27], although *SND3 *has not consistently been detected as a VND6/VND7 direct target. *SND2 *is indirectly regulated by SND1/NST1 [21,28], but there exists no evidence for regulation by VND6/VND7. Loss- and gain-of-function mutagenesis of *SND2*, but interestingly also that of *SND3 *and *MYB103*, produced a fibre-specific phenotype [21]. Whilst dominant repression [29] drastically reduced fibre-specific SCW thickness, individual overexpression of *MYB103*, *SND2 *and *SND3 *increased SCW thickness in interfascicular and xylary fibres, with no apparent impact on vessels. In stems a reduction in glucose, xylose and mannose cell wall sugars occurred during dominant repression of *MYB103*, *SND2 *or *SND3*. Conversely, all three TFs could transactivate the SCW cellulose-associated *CesA8 *gene promoter, but not representatives of hemicellulose (*IRX9*) or lignin (*4CL1*) biosynthesis [21].

The regulation and function of *SND2 *may differ in herbaceous and woody plants, especially in woody tissues which possess greater proportions of fibre cells than stems of herbaceous plants. This may be facilitated by gene family expansion and specialization in woody plants [30]. As many as four putative *SND2 *orthologs exist in poplar due to significant expansion of the NAC family [31], some paralogs of which may have undergone subfunctionalization in *Populus *[32]. All four putative orthologs were found to be preferentially expressed in developing xylem and phloem fibres [31]. Overexpression of one of the putative orthologs, *PopNAC154*, resulted in a decrease in height and an increase in the proportion of bark to xylem in poplar trees, with no perceptible effect on SCW thickness [31]. This apparent conflict with the *SND2 *overexpression phenotype in *Arabidopsis *[21] illustrates that the regulatory function of SND2 homologs may differ between herbaceous and woody plants.

The observation that *SND2 *overexpression led to enhanced SCW formation in *Arabidopsis *fibres and that it potentially regulates cellulosic genes are important findings, because evidence supports the existence of a similar transcriptional network regulating fibre SCW development in angiosperm trees [13,33,34]. However, several aspects of the biological function of *SND2 *remain to be resolved before the biotechnological potential of the gene can be determined. The global targets of SND2 have not been identified and its position in the transcriptional network has not been established. The finding that SND2 regulates cellulose, but not xylan and lignin biosynthetic genes, was based on a single representative gene from each pathway [21]. A greater knowledge of SND2 targets is required to confidently negate its regulation of hemicellulose and lignin biosynthesis. It is also unclear from the analysis by Zhong *et al. *[21] whether *SND2 *overexpression invariably leads to increased fibre SCW thickness, both in *Arabidopsis *and in woody taxa. Finally, the effect of *SND2 *overexpression on cell wall chemistry has not yet been reported.

We aimed to further characterize the position and regulatory role of *SND2 *in the fibre SCW transcriptional network, and confirm the phenotypic effects of *SND2 *overexpression in *Arabidopsis *and *Eucalyptus *plants. Our objectives were to identify genes that are differentially expressed in *SND2-*overexpressing plants, and determine the overall effect on *Arabidopsis *development and biomass production, as well as fibre SCW formation in *Arabidopsis *and *Eucalyptus*. We describe novel regulatory roles for SND2 in fibre SCW development, and propose a model for the role of SND2 in the transcriptional network regulating SCW formation.

## Results

### Whole-transcriptome expression profiling of *SND2*-overexpressing *Arabidopsis *plants

SND2 was previously shown to transactivate the *CesA8 *gene promoter in *Arabidopsis *protoplasts [21]. In order to identify other genes regulated by SND2 *in planta*, we overexpressed *SND2 *in *Arabidopsis *plants by cloning the *SND2 *coding sequence into the overexpression vector pMDC32 [35]. We introduced the construct into *A. thaliana *Col-0 plants and randomly selected three homozygous T4 lines (A, B, and C), from a pool of T1 transgenic plants herein denoted "SND2-OV". We confirmed that *SND2 *was strongly upregulated in the T4 SND2-OV lines using RT-qPCR analysis (Additional file 1, Figure S1). We then tested the T4 SND2-OV lines for preliminary evidence of *CesA8 *upregulation in lower inflorescence stems using RT-qPCR analysis. Interestingly, line A ("SND2-OV(A)") exclusively showed evidence for *CesA8 *upregulation (not shown), and was therefore selected for transcriptome analysis.

In order to determine which genes were differentially expressed as a result of *SND2 *overexpression in *Arabidopsis *stems, the transcriptome of SND2-OV(A) plants was compared to that of the wild type with respect to the bottom 100 mm of primary inflorescence stems. High quality total RNA (RQI > 9.3) was isolated from three biological replicates of eight-week-old wild type and SND2-OV(A) plant stems, and labelled cDNA hybridized to Agilent 4 × 44k *Arabidopsis *transcriptome arrays. Significantly differentially expressed genes (DEG) were identified as those with an experiment-wise false discovery rate below 0.05 and fold change > |±1.5|. This analysis identified a total of 155 upregulated and 68 downregulated genes in SND2-OV(A) relative to the wild type (Additional file 2).

In order to identify overrepresented gene ontology (GO) classes amongst the DEGs, the GOToolBox resource [36] was interrogated with a hypergeometric test (Benjamini and Hochberg correction) using The *Arabidopsis *Information Resource [37] annotation set. Significantly enriched biological processes (*P *< 0.01) revealed a predominant role of the DEGs in (secondary) cell wall organization and biogenesis, carbohydrate metabolism, signalling and response to stimulus (Additional file 3, Table S1).

### Identification of putative SND2 targets

*SND2 *is preferentially expressed in xylem [21,38]. We hypothesized that targets of SND2 would be co-expressed with endogenous *SND2 *transcripts. The tissue-specific expression of DEGs identified in SND2-OV(A) (fold change > |±1.5|) was explored by observing the expression patterns across selected *Arabidopsis *tissues using the Genevestigator V3 [39] anatomy clustering tool. At the time of analysis, the Genevestigator database totalled 374 publicly available microarray studies for *Arabidopsis*, encompassing 6290 samples. Of 223 genes in our SND2-OV(A) dataset, 190 were represented by unique probe sets on high quality ATH1 22k arrays. We examined the endogenous expression of these genes across 26 tissues based on results from 4422 arrays, and subjected the genes to hierarchical clustering according to their absolute expression profiles. The majority of genes did not conform to a single expression pattern, with only ~9% of the genes displaying expression profiles clearly resembling that of native *SND2 *transcript, i.e. with preferential expression in SCW-containing tissues (Additional file 1, Figure S2). Thus, the majority of genes differentially expressed as a result of *SND2 *overexpression were not generally associated with SCW-containing tissues.

Novel targets arising from ectopic overexpression of cell wall-associated NAC TFs have been reported previously [40]. It is possible that a similar phenomenon occurred in our study, since the bulk of the sampled transgenic stems comprised tissues where *SND2 *is not naturally expressed. This may explain the small proportion of DEGs that were co-expressed with *SND2 *in Additional file 1, Figure S2. To avoid this possibility, we stringently defined the putative authentic targets of *SND2 *as those that were also a subset of SND1-regulated genes, the latter identified by microarray analysis of *SND1*-overexpressing *Arabidopsis *plants by Ko *et al. *[28]. The age of the plants in the cited study (~8.5 weeks) and the tissue sampled (lower 50 mm of the inflorescence stem) was similar to our experiment. *SND2*, a known indirect target of SND1 [21], was the most strongly upregulated TF in the SND1-overexpressing plant stems [28], further justifying our approach.

We extracted genes common to the Ko *et al. *[28] data and our significant SND2-OV(A) microarray data, without fold-change filtering. Seventy five genes were shared between the two datasets, herein denoted "SND2∩Ko", ~79% of which were regulated in a consistent direction (Table 1). Amongst them, genes involved in transcription, (secondary) cell wall biosynthesis, cell wall expansion and modification, carbohydrate metabolism, stress response and proteins of unknown function were prominent (Table 1). There was notably no differential expression of monolignol biosynthetic genes.

**Table 1:** Subset of SND1-regulated genes [28] also significantly differentially expressed in stems of eight-week-old SND2-OV(A) plants relative to wild type (SND2∩Ko).

Locus	Description	Fold change	*P*-value^a^
**Transcription^b^**			
AT4G28500	ANAC073/SND2 (*Arabidopsis *NAC domain containing protein 73); transcription factor	> 100.00^c^	0.00E+00
AT1G63910	MYB103 (MYB DOMAIN PROTEIN 103); DNA binding/transcription factor	1.83	1.13E-11
AT1G52890	ANAC019 (*Arabidopsis *NAC domain containing protein 19); transcription factor	1.44	3.07E-04
AT1G32770	ANAC012/NST3/SND1 (*ARABIDOPSIS *NAC DOMAIN CONTAINING PROTEIN 12); transcription factor	1.42	6.93E-04
AT4G17245	Zinc finger (C3HC4-type RING finger) family protein	1.36	4.97E-03
AT5G13330	RAP2.6L (related to AP2 6L); DNA binding/transcription factor	1.36	4.87E-03
**Secondary cell wall biosynthesis and cell wall modification**			
AT2G03090	EXPA15 (EXPANSIN A15)	2.03	3.60E-16
AT5G44030	CESA4 (CELLULOSE SYNTHASE 4); transferase, transferring glycosyl groups	1.84	7.91E-12
AT5G60490	FLA12 (fasciclin-like arabinogalactan-protein 12)	1.83	1.37E-11
AT2G38080	IRX12/LAC4 (laccase 4); copper ion binding/oxidoreductase	1.77	2.51E-10
AT5G17420	CesA7/IRX3 (IRREGULAR XYLEM 3, MURUS 10); cellulose synthase	1.73	1.25E-09
AT5G03170	FLA11 (fasciclin-like arabinogalactan-protein 11)	1.72	2.80E-09
AT5G03760	CSLA09 (RESISTANT TO AGROBACTERIUM TRANSFORMATION 4); transferase, transferring glycosyl groups	1.66	4.33E-08
AT4G18780	CESA8 (CELLULOSE SYNTHASE 8); cellulose synthase/transferase, transferring glycosyl groups	1.63	1.33E-07
AT5G15630	COBL4/IRX6 (COBRA-LIKE4)	1.62	2.12E-07
AT5G60020	LAC17 (laccase 17); copper ion binding/oxidoreductase	1.59	7.44E-07
AT3G18660	PGSIP1 (PLANT GLYCOGENIN-LIKE STARCH INITIATION PROTEIN 1); transferase, transferring glycosyl groups	1.58	1.21E-06
AT3G50220	IRX15; domain of unknown function 579 (DUF579)-containing protein	1.55	3.97E-06
AT5G54690	GAUT12/IRX8/LGT6 (GALACTURONOSYLTRANSFERASE 12); polygalacturonate 4-alpha-galacturonosyltransferase	1.39	1.81E-03
AT5G59290	UXS3 (UDP-GLUCURONIC ACID DECARBOXYLASE)	1.38	2.66E-03
AT1G19300	GATL1/GLZ1/PARVUS (GALACTURONOSYLTRANSFERASE-LIKE 1); polygalacturonate 4-alpha-galacturonosyltransferase	1.33	1.08E-02
AT5G01360	TBL3; domain of unknown function 231 (DUF231)-containing protein	1.31	2.08E-02
AT1G27440	GUT2/IRX10 (glucuronoxylan glucuronosyltransferase)	1.30	2.57E-02
**Signal transduction**			
AT3G16920	CTL2 (Chitinase -like protein 2)	1.71	4.23E-09
AT1G09440	Protein kinase family protein	1.47	1.21E-04
AT3G15050	IQD10 (IQ-domain 10); calmodulin binding	1.46	1.68E-04
AT1G27380	RIC2 (ROP-INTERACTIVE CRIB MOTIF-CONTAINING PROTEIN 2)	1.43	4.07E-04
AT1G56720	Protein kinase family protein	1.41	9.28E-04
AT1G08340	Rho GTPase activating protein, putative	1.38	2.29E-03
AT2G36570	Leucine-rich repeat transmembrane protein kinase, putative	1.32	1.47E-02
**Carbohydrate metabolism**			
AT5G35740	Glycosyl hydrolase family protein 17	1.57	2.54E-06
AT1G04680	Pectate lyase family protein	1.57	2.11E-06
AT4G36360	BGAL3 (beta-galactosidase 3); beta-galactosidase	1.45	2.66E-04
AT1G19940	GH9B5 (GLYCOSYL HYDROLASE 9B5); hydrolase, hydrolyzing O-glycosyl compounds	1.41	1.09E-03
**Abiotic and biotic stress response**			
AT5G42180	Peroxidase 64 (PER64) (P64) (PRXR4)	1.66	4.29E-08
AT1G72060	Serine-type endopeptidase inhibitor	1.52	1.59E-05
AT4G27410	RD26 (RESPONSIVE TO DESSICATION 26)	1.37	3.20E-03
AT2G37130	Peroxidase 21 (PER21) (P21) (PRXR5)	1.29	3.47E-02
AT4G23690	Disease resistance-responsive family protein/dirigent family protein	-1.34	7.80E-03
AT1G68850	Peroxidase, putative	-1.41	1.08E-03
AT4G11650	OSM34 (OSMOTIN 34)	-1.69	8.89E-09
AT5G24780	VSP1 (VEGETATIVE STORAGE PROTEIN 1); acid phosphatase	-2.38	3.08E-24
**Cytoskeleton**			
AT1G50010	TUA2 (tubulin alpha-2 chain)	1.47	1.26E-04
AT5G23860	TUB8 (tubulin beta-8)	1.36	4.58E-03
**One-carbon metabolism**			
AT3G23810	SAHH2 (S-ADENOSYL-L-HOMOCYSTEINE (SAH) HYDROLASE 2); adenosylhomocysteinase	1.41	9.68E-04
**Lipid metabolism**			
AT1G29670	GDSL-motif lipase/hydrolase family protein	1.28	4.39E-02
AT1G21360	GLTP2 (GLYCOLIPID TRANSFER PROTEIN 2)	-1.77	1.99E-10
**Wax biosynthesis**			
AT1G02205	CER1 (ECERIFERUM 1)	1.35	2.04E-03
**Unknown function**			
AT3G22540	Unknown protein	1.58	1.50E-06
AT1G33800	Unknown protein	1.55	4.24E-06
AT4G27435	Unknown protein	1.43	5.42E-04
AT5G64190	Unknown protein	1.42	6.86E-04
AT5G61340	Unknown protein	1.39	1.63E-03
AT1G07120	Unknown protein	1.32	1.47E-02
AT1G03820	Unknown protein	1.32	1.88E-02
AT1G24600	Unknown protein	-1.33	1.29E-02
AT5G66170	Unknown protein	-1.36	4.29E-03
**Unassigned**			
AT1G55330	AGP21 (ARABINOGALACTAN PROTEIN 21)	1.71	4.63E-09
AT4G28050	TET7 (TETRASPANIN7)	1.64	1.16E-07
AT3G54040	Photoassimilate-responsive protein-related	1.62	2.52E-07
AT2G41250	Haloacid dehalogenase-like hydrolase superfamily protein	1.57	2.23E-06
AT5G44130	FLA13 (FASCICLIN-LIKE ARABINOGALACTAN PROTEIN 13 PRECURSOR)	1.44	3.97E-04
AT2G05540	Glycine-rich protein	1.43	4.36E-04
AT3G62020	GLP10 (GERMIN-LIKE PROTEIN 10); manganese ion binding/metal ion binding/nutrient reservoir	1.40	1.46E-03
AT5G10430	AGP4 (ARABINOGALACTAN-PROTEIN 4)	1.38	2.29E-03
AT3G52370	FLA15 (FASCICLIN-LIKE ARABINOGALACTAN PROTEIN 15 PRECURSOR)	1.37	4.03E-03
AT2G05380	GRP3S (GLYCINE-RICH PROTEIN 3 SHORT ISOFORM)	1.37	3.58E-03
AT2G22170	Lipid-associated family protein	1.35	7.62E-03
AT1G72230	Plastocyanin-like domain-containing protein	1.32	1.70E-02
AT4G04460	Aspartyl protease family protein	-1.29	4.11E-02
AT1G76790	O-methyltransferase family 2 protein	-1.39	1.94E-03
AT3G28220	Meprin and TRAF homology domain-containing protein/MATH domain-containing protein	-1.60	5.82E-07
AT4G25010	Nodulin MtN3 family protein	-1.64	1.00E-07
AT2G39030	GCN5-related N-acetyltransferase (GNAT) family protein	-1.80	4.90E-11
AT5G09530	Hydroxyproline-rich glycoprotein family protein	-3.09	1.31E-41

^a^Adjusted *P*-value according to False Discovery Rate (FDR) method

^b^Genes are categorized by Gene Ontology classification according to The *Arabidopsis *Information Resource www.arabidopsis.org, unless otherwise described in the main text.

^c^Transgene. The fold change is likely an underestimate of the actual value because this target displayed a saturated hybridization signal

We independently assessed the possible function of *SND2 *by identifying genes co-expressed with native *SND2 *transcript from the AtGenExpress Plus Extended Tissue Set public microarray data using Expression Angler [41], employing a stringent Pearson correlation coefficient threshold (R > 0.90). Genes associated with SCW biosynthesis (e.g. secondary wall *CesAs*, *IRX *genes) as well as TFs previously implicated in SCW regulation (*MYB103*, *SND1*), were amongst the 31 genes found to be co-expressed with *SND2 *(Table 2), supporting a role of *SND2 *in SCW regulation. 22 of the genes were differentially expressed in the SND2∩Ko data (Table 2).

**Table 2:** Genes tightly co-expressed with endogenous *SND2 *transcript.

Co-expressed gene	R-value	Description	SND2∩Ko
PGSIP1 (AT3G18660)	0.980	Plant glycogenin-like starch initiation protein 1	√
IQD10 (AT3G15050)	0.979	Calmodulin-binding protein	√
MYB103 (AT1G63910)	0.973	Secondary cell wall-associated transcription factor	√
IRX8 (AT5G54690)	0.972	Galacturonosyltransferase 12	√
COBL4 (AT5G15630)	0.972	COBRA-like protein	√
IRX15 (AT3G50220)	0.967	DUF579 protein required for normal xylan synthesis	√
IRX15-L (AT5G67210)	0.963	DUF579 protein required for normal xylan synthesis	
CesA7 (AT5G17420)	0.962	Secondary cell wall cellulose synthase protein	√
GLP10 (AT3G62020)	0.959	Germin-like protein 10	√
FLA11 (AT5G03170)	0.958	Fasciclin-like arabinogalactan protein	√
LAC4 (AT2G38080)	0.955	IRREGULAR XYLEM 12	√
LAC2 (AT2G29130)	0.953	Laccase	
AT1G08340	0.952	Rho GTPase activating protein	√
CTL2 (AT3G16920)	0.951	Chitinase-like protein 2	√
AT1G80170	0.950	Pectin lyase-like superfamily protein	
AT2G41610	0.950	Unknown protein	
SND1 (AT1G32770)	0.948	Secondary cell wall-associated transcription factor	√
RIC2 (AT1G27380)	0.948	ROP-interactive CRIB motif-containing protein	√
MAP65-8 (AT1G27920)	0.941	Microtubule-associated protein	
AT1G07120	0.938	Unknown protein	√
AT2G31930	0.936	Unknown protein	
CesA4 (AT5G44030)	0.934	Secondary cell wall cellulose synthase protein	√
AT4G27435	0.934	Protein of unknown function (DUF1218)	√
AT1G22480	0.933	Cupredoxin superfamily protein	
IRX10 (AT1G27440)	0.929	Glucuronoxylan glucuronosyltransferase	√
CesA8 (AT4G18780)	0.926	Secondary cell wall cellulose synthase protein	√
RWA3 (AT2G34410)	0.915	Polysaccharide O-acetyltransferase	
AT4G28380	0.915	Leucine-rich repeat (LRR) family protein	
LAC17 (AT5G60020)	0.914	Laccase	√
PARVUS (AT1G19300)	0.904	Polygalacturonate 4-α-galacturonosyltransferase	√
TBL3 (AT5G01360)	0.902	DUF231 protein involved in cellulose biosynthesis	√

Expression Angler [41] was used to find co-expressed genes in the AtGenExpress Plus Extended Tissue Set microarray data. The R-value represents the Pearson correlation coefficient of co-expression, set to a threshold of R > 0.90. Co-expressed genes that were also differentially expressed in the SND2∩Ko subset of *SND2 *overexpression data (Table 1) are indicated in the far right column.

**Figure 1:**
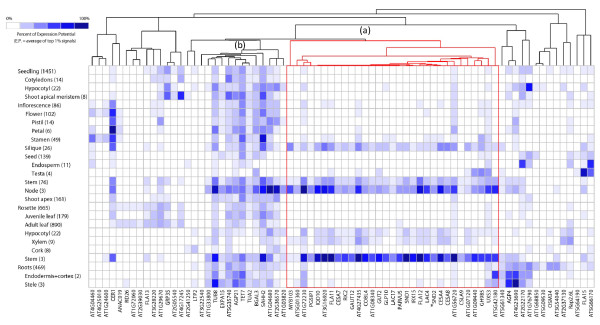
**Absolute transcript abundance of SND2∩Ko genes represented on ATH1 22k arrays in *Arabidopsis *tissues and organs**. Genevestigator V3 [39] was used for microarray data mining, and the anatomical cluster analysis tool was used to visualize and cluster the genes according to their tissue-specific expression patterns. Tissues/organs are staggered hierarchically, and the number of arrays on which the data are based is indicated in parentheses. Absolute transcript values are expressed as a percentage of their expression potential (E.P.), where E.P. is the mean of the top 1% of hybridization signals for a given probe set across all arrays. Cluster (a), highlighted in red, is comprised of 31 genes, including *SND2 *(*), which displayed preferential expression in tissues and organs where *SND2 *is expressed. Cluster (b) encompasses of 13 genes which displayed preferential expression in inflorescence stems and nodes, rosette stems, and in some cases the stamen, seedling hypocotyl and/or vasculature (stele) of roots.

The seventy five SND2∩Ko genes represented on the ATH1 22k array were subjected to hierarchical clustering across the Genevestigator V3 *Arabidopsis *anatomy database [39] as before to analyze their tissue specificity. Unique probe sets were found for all but one gene (AT5G24780). One cluster (a) contained 31 genes preferentially expressed in a similar fashion to *SND2*, namely in inflorescence nodes and stem, rosette stem and xylem, and silique (Figure 1). Another cluster of 13 genes (b) appeared to exhibit preferential expression in inflorescence stems and nodes, rosette stems, and occasionally seedling hypocotyls, root steles and anther-containing stamens, all of which contain SCWs to some degree. Thus, compared to the original SND2-OV(A) dataset, a much higher percentage (59%) of genes in the SND2∩Ko dataset displayed preferential expression in tissues containing SCWs. Combined with the AtGenExpress co-expression analysis, these data support the role of *SND2 *in SCW regulation and the validity of the SND2∩Ko dataset as the most likely direct or indirect targets of SND2.

**Figure 2:**
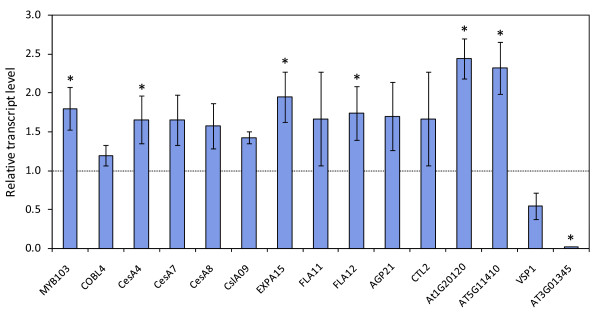
**RT-qPCR analysis of selected genes differentially expressed in inflorescence stems of eight-week-old SND2-OV(A) and wild type plants**. SND2-OV(A) plants were grown alongside the wild type in three biological replicate pairs, with primary stems from six plants pooled per sample. SND2-OV(A) transcript levels were normalized to the wild type in each replicate (assigned a value of 1, for each gene), hence error bars indicate the standard error of the deviation from wild type across biological replicates. Significance was evaluated by a one-tailed paired t-test, in accordance with the expected direction of response for each gene; *P < 0.05.

The microarray results were validated by RT-qPCR analysis. We profiled fifteen genes based on the microarray RNA isolated from stems of eight-week-old SND2-OV(A) and wild type plants (Figure 2). All RT-qPCR profiles agreed with the microarray data, and seven genes were significantly (*P *< 0.05) upregulated (including *CesA4*, *EXPA15*, *FLA12 *and *MYB103*). We also confirmed that the endogenous *SND2 *transcript showed no significant change in SND2-OV(A) stems, whereas total *SND2 *transcript abundance (the sum of transgenic and endogenous transcripts) in SND2-OV(A) stems was ~180-fold that of the wild type (not shown). We obtained similar results for selected genes from plants grown in an independent trial (Additional file 1, Figure S3).

We were interested in the temporal effect of inflorescence stem development on the putative targets of SND2 when constitutively expressed. We therefore performed a second microarray analysis of SND2-OV(A) and wild type plants at four weeks of age, sampling inflorescence stems that were ~120 mm tall. Of the 21 upregulated and 24 downregulated DEGs, no SND2∩Ko candidates were present, nor were any SCW biosynthesis-associated genes (Additional file 4). This result suggests that an additional co-regulator(s), only expressed after four weeks, is required for SND2 to function in fibre SCW regulation.

### Effect of *SND2 *overexpression on *Arabidopsis *SCW thickness, biomass and SCW composition

**Figure 3:**
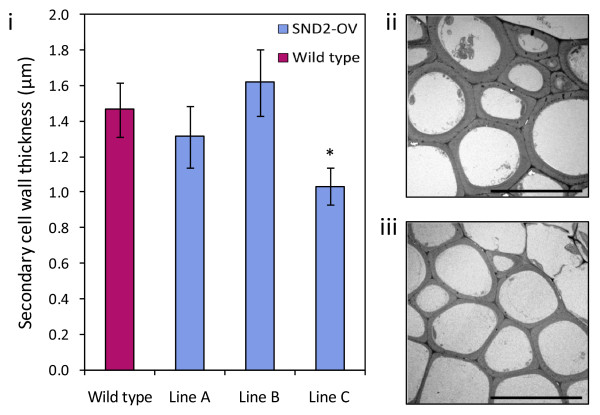
**(i) SCW thickness in IFs of eight-week-old wild type and T4 homozygous SND2-OV lines A, B and C**. Measurements are based on scanning electron micrographs. Error bars indicate the standard error of the mean of three biological replicates (21-42 fibres were measured per line). *Significantly different from wild type according to homoscedastic two-tailed Student's t-test (*P *< 0.02). Transmission micrographs of representative IF regions of wild type and SND2-OV line C stems are shown in **(ii) **and **(iii) **respectively (scale bars = 20 μm).

Zhong *et al. *[21] previously reported that *SND2 *overexpression significantly increased SCW thickness in interfascicular fibres (IFs) of *Arabidopsis *inflorescence stems. However, amongst our homozygous SND2-OV lines, scanning electron microscopy (SEM) revealed no significant changes in fibre wall thickness for lines A and B, whilst line C had significantly thinner SCWs than the wild type (Figure 3). These results were reproduced in an independent trial using light microscopy (Additional file 1, Figure S4).

**Figure 4:**
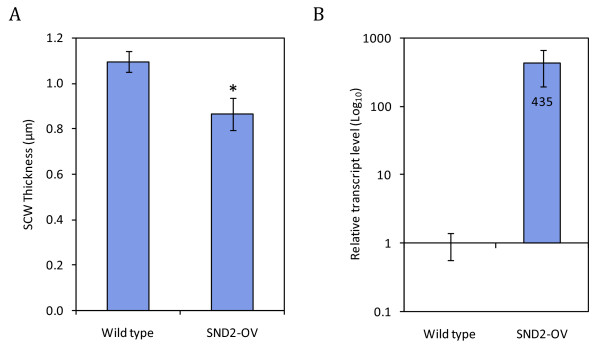
**Effect of *SND2 *overexpression on IF wall thickness in T1 generation stems**. **(A) **Mean SCW thickness in IFs of eight-week-old wild type and T1 generation SND2-OV stems. Representative light microscopy images are shown in Additional file 1, Figure S5. Error bars indicate the standard error of the mean of eight wild type and seven T1 plants (26-48 fibres were measured per plant). *Significantly different from wild type based on homoscedastic two-tailed Student's t-test (*P *< 0.02). **(B) **Corresponding transcript abundance of total *SND2 *transcript in lower stems of six wild type and six SND2-OV T1 plants used for SCW measurements, as measured by RT-qPCR. The primer pair quantifies endogenous and transgenic *SND2 *transcript. Total *SND2 *transcript is ~435-fold relative to the wild type, represented here on a log_10 _scale. Calibrated Normalized Relative Quantity (CNRQ) values were obtained by normalization against three control genes. Error bars indicate the standard error of the mean of six plants.

Fibre SCW thickness was additionally assessed in lower inflorescence stems of seven T1 SND2-OV and eight wild type plants using light microscopy. Representative micrographs are shown in Additional file 1, Figure S5. The T1 lines manifested a significant (21%, *P *< 0.02) decrease in mean IF SCW thickness (Figure 4A) that resembled SND2-OV line C and the *SND2 *dominant repression phenotype reported previously [21]. Combined endogenous and transgenic *SND2 *transcript abundance from T1 plants exceeded that of the wild type plants by ~435-fold, ruling out co-suppression as an explanation for the phenotype (Figure 4B). Although no significant correlation could be found between *SND2 *transcript abundance and SCW thickness, our data confirm that strong *SND2 *overexpression reduces IF SCW thickness.

We hypothesized that *SND2 *overexpression could influence overall inflorescence stem biomass, irrespective of IF SCW thickness. The entire inflorescence stems of eight-week-old T4 SND2-OV lines A, B and C were weighed to determine total biomass yield. Only in the most highly overexpressing line, SND2-OV line C, was biomass significantly different from the wild type, where fresh and dry biomass was decreased (Additional file 1, Figure S6). This was despite the fact that all SND2-OV lines appeared phenotypically normal and exhibited no stunting or dwarfing (results not shown). Biomass profiles in Additional file 1, Figure S6 were in agreement with the IF SCW thickness profile for each respective line (Figure 3), suggesting a direct relationship between IF SCW thickness and biomass yield, and therefore a negative effect of excessive *SND2 *overexpression on biomass yield.

The chemical composition of the inflorescence stems was investigated by Klason lignin analysis and quantification of monosaccharides following complete acid hydrolysis. SND2-OV(A) exhibited a nominal but statistically significant 2.5% relative decrease in total lignin (Table 3, *P *= 0.03). This was likely due to a reduction in insoluble lignin (Table 3). No changes were apparent in the relative abundance of glucose and xylose, and only mannose and rhamnose were significantly increased in line A (*P *< 0.05) by 7.4% and 5.4% respectively (Table 4). We also quantified the chemical composition of SND2-OV line C to investigate SCW composition when fibre wall thickness was reduced. However, no change in lignin or monosaccharide content was detected against the wild type (not shown).

**Table 3:** Klason lignin content of SCW material of T4 SND2-OV(A) stems compared to the wild type control.

Sample	Total lignin (%)	Insoluble lignin (%)	Soluble lignin (%)
SND2-OV(A)	21.06 ± 0.18	15.74 ± 0.20	5.32 ± 0.03
Wild type	21.61 ± 0.21	16.25 ± 0.32	5.43 ± 0.18
*P*-value	0.033	0.074	0.623

Values are expressed as the mean of three biological replicates plus or minus the standard error of the mean. *P*-values are based on paired two-tailed Student's t-tests between SND2-OV(A) and the wild type.

**Table 4:** Monosaccharide composition of SCW material of T4 SND2-OV(A) stems compared to the wild type control.

Sample	Glucose	Xylose	Mannose	Galactose	Arabinose	Rhamnose	Fucose
SND2-OV(A)	343.36 ± 1.42	109.85 ± 0.16	18.06 ± 0.04	18.59 ± 0.08	8.13 ± 0.03	12.83 ± 0.02	0.84 ± 0.01
Wild Type	344.94 ± 2.92	108.62 ± 2.71	16.82 ± 0.35	18.25 ± 0.41	7.88 ± 0.09	12.17 ± 0.18	0.88 ± 0.01
*P*-value	0.561	0.663	0.049	0.563	0.238	0.023	0.663

Values (mg/g dry weight) are expressed as the mean of three biological replicates plus or minus the standard error of the mean. *P*-values are based on paired two-tailed Student's t-tests between SND2-OV(A) and the wild type.

### Induced somatic overexpression of *SND2 *in *Eucalyptus *stem sectors

Compared to herbaceous annuals such as *Arabidopsis*, woody perennials devote a larger proportion of carbon allocation to xylem formation. We therefore examined the effect of *Arabidopsis SND2 *overexpression on xylem fibre characteristics in *Eucalyptus *trees by Induced Somatic Sector Analysis [42]. Stems were transformed with a pCAMBIA1305.1 construct (containing the *β*-glucuronidase or 'GUS' reporter gene) overexpressing *SND2*. Tree stems were harvested after 195-210 days, transgenic sectors were identified in the cross-sections via GUS reporter staining, and etched to delineate the transgenic sectors prior to SEM analysis (Additional file 1, Figure S7).

Fibre dimensions were measured from SEM micrographs as a percentage change between eleven transgenic sectors and adjacent wild type sectors for the *SND2-*overexpressing gene construct, as well as nine empty vector control (EVC) sectors expressing only the GUS reporter. Fibre cell area (i.e. average fibre cross-section area) was significantly increased in SND2-OV sectors compared to EVC sectors (Table 5, *P *= 0.042), demonstrating that *SND2 *influences fibre development in *Eucalyptus*. Fibre cell wall area and lumen area, which comprise fibre cell area, were marginally increased in *SND2*-overexpressing sectors relative to EVC sectors, but the differences were not statistically significant for these individual parameters. However, since the increase in cell wall area in *SND2-*overexpressing sectors was close to significant (*P *= 0.066), it is reasonable to suggest that the increase in fibre cell area was mainly due to a cell wall area increase rather than a lumen area contribution. Measurement of fibre cell area in the *Arabidopsis *T4 and T1 SND2-OV lines revealed no significant differences relative to the wild type (not shown).

**Table 5:** Change in fibre SCW thickness, cell wall area, fibre cell area and lumen area of *Eucalyptus *sectors overexpressing *Arabidopsis SND2*.

Sample	Cell wall thickness (%)	Cell wall area (%)	Fibre cell area (%)	Lumen area (%)
SND2-OV	9.99 ± 2.34	14.60 ± 2.64	14.41 ± 2.44	9.68 ± 4.65
EVC	5.54 ± 4.33	6.16 ± 4.98	5.04 ± 4.84	3.78 ± 7.13
*P*-value	0.177	0.066	0.042	0.241

*SND2-*overexpressing (SND2-OV) and empty vector control (EVC) sector values are expressed as a percentage change relative to non-transformed tissues. Measurements were obtained from 11 (SND2-OV) and 9 (EVC) transgenic-nontransgenic control sector pairs from two F1 *Eucalyptus *hybrids. *P *values are based on one-tailed Student's t-test.

## Discussion

A role for *SND2 *in regulating *Arabidopsis *fibre SCW formation was previously suggested by studies establishing it as an indirect target of SND1, a master regulator of fibre SCW development [18,20-22,28]. In promoter transactivation experiments, *SND2 *was implicated in the regulation of cellulose (*CesA8*), but a role in regulating hemicellulose or lignin biosynthesis seemed unlikely [21]. A particularly interesting finding was a fibre cell-specific increase in SCW thickness when *SND2 *was constitutively overexpressed, mirrored by decreased fibre SCW thickness in dominant repression lines [21]. The proposed role of SND2 in *Arabidopsis *fibre SCW formation has not been independently validated and the full suite of genes regulated by SND2 has not been elucidated. To address this, we performed microarray analysis on a homozygous *SND2 *overexpressing line, SND2-OV(A), which also exhibited significant upregulation of the *CesA8 *gene.

TFs have been shown to activate novel targets when ectopically expressed. A striking example was described by Bennett *et al. *[40] for NAC TFs regulating primary cell wall modification in the root cap. Overexpression in stems caused ectopic lignification and ectopic expression of SCW genes [40]. Our microarray results therefore likely include direct and indirect targets of SND2, as well as genes misregulated due to the ectopic overexpression of *SND2*. To discriminate native targets of SND2, we defined a subset of genes (SND2∩Ko) regulated by SND1 [28] that were also found to be differentially expressed in this study (Table 1). We reasoned that obtaining the SND1 subset of targets would be a robust approach for reducing ectopic noise, because *SND2 *is indirectly, but strongly activated by SND1 [21,28] and native SND2 targets should therefore be a subset of the SND1 targets. Further support for defining these seventy five genes as putative SND2 targets was provided by the finding that a large proportion (71%) of genes co-regulated (R > 0.9) with *SND2 *in a large compendium of AtGenExpress microarray experiments were included in the SND2∩Ko set (Table 2). Recently, Zhong *et al. *[43] demonstrated transactivation of poplar *CesA4*, *CesA8*, GT43 and GT47 family gene promoters by a poplar co-ortholog of *SND2*, providing a third line of evidence that *SND2 *regulates SCW-associated genes.

The SND2∩Ko set (Table 1) prominently included genes involved in SCW biosynthesis, transcriptional regulation and signalling. Amongst the SCW-associated genes, *CesA4*, *CesA7 *and *CesA8 *are involved in SCW cellulose biosynthesis [44-46]. *COBL4 *and its orthologs also appear to be involved in SCW cellulose formation [47,48], and recently a homolog of *TRICHOME BIREFRINGENCE*, *TBL3 *(AT5G01360), was shown to affect secondary cellulose deposition and possibly SCW structure through alterations in pectin methylesterification [49]. *PARVUS*, *IRX8 *and *IRX10 *are required for xylan biosynthesis in SCWs [50-53]. *IRX15 *and *IRX15L*, encoding functionally redundant DUF579 proteins, were recently shown to be essential for normal xylan biosynthesis [54,55], but only the former was upregulated in SND2-OV(A) stems (Table 1). *PGSIP1 *and *UXS3 *are co-expressed with xylan synthases, with good evidence supporting a xylan α-glucuronosyltransferase function for *PGSIP1 *[56] and a UDP-xylose synthase function for *UXS3 *[57,58]. As shown previously [21], *SND2 *did not activate the xylan-associated *IRX9 *gene in this study, nor did it activate lignin-associated *4CL1*. *LAC4 *and *LAC17 *encode laccases, an enzyme group that has been linked to SCW lignin polymerization [59]. *LAC4 *and *LAC17 *are regulated by lignin-specific TFs MYB58 and MYB63 [60] and were also recently shown to affect lignification in *Arabidopsis *xylem, with *LAC17 *specifically implicated in G-lignin polymerization in IFs [61]. Our results (Table 1) thus suggest an additional role for *SND2 *in the regulation of lignification distinct from that of monolignol biosynthesis.

Several TFs were upregulated in the SND2∩Ko set (Table 1). ANAC019 regulates biotic and abiotic stress responses [62,63]. AT4G17245 is a C3HC4 RING-type zinc finger gene of unknown function. However, at least one C3HC4 gene, AT1G72220, has been previously implicated in SCW formation [47]. We observed upregulation of *RAP2.6L*, an ethylene response factor involved in shoot regeneration and abiotic stress response [64,65]. The upregulation of *SND1 *and *MYB103 *in SND2(OV) plants was unexpected. SND1, a master activator of SCW biosynthesis in fibres [18,20-22,28], is expected to be upstream of SND2 in the transcriptional network. It also seems intuitive that *SND2 *acts downstream of *MYB103*, since *SND2 *is an indirect target of SND1, whilst *MYB103 *is a direct target of *SND1 *[21]. A positive feedback loop may exist through which upregulation of *SND2*, or another TF (Table 1), promotes *SND1 *expression.

**Figure 5:**
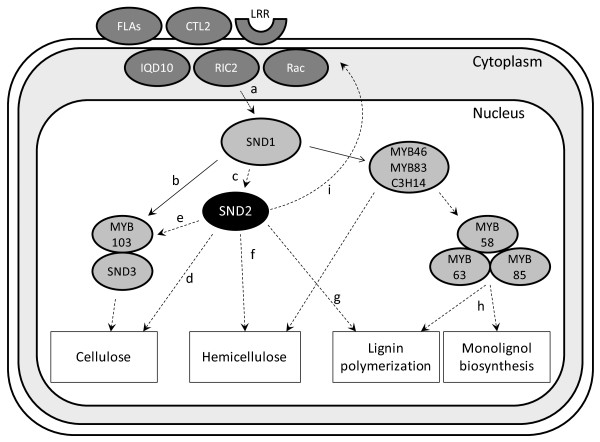
**Proposed model of SND2-mediated SCW regulation in IFs**. Solid lines indicate known direct protein-DNA interactions. Dashed lines indicate direct or indirect protein-DNA interactions. Master regulator SND1 is activated by a signal transduction pathway proposed by Oikawa *et al. *[56] (a). SND1 directly activates transcription of *MYB103 *and *SND3 *(b), and indirectly activates *SND2 *through an unknown intermediate (c; [21]). SND2 activates cellulose-synthesizing *CesA*s, either directly (d) or through the activation of *MYB103 *(e), which is known to activate SCW cellulose gene, *CesA8 *[21]. SND2 regulates hemicellulosic genes (f; Table 1), independently to a similar role played by direct SND1 targets *MYB46*, *MYB83 *or *C3H14 *[76-78]. SND2 plays a role in lignification through activation of lignin polymerization genes *LAC4 *and *LAC17 *(g; Table 1), but it does not regulate monolignol biosynthetic genes as is the case for MYB58, MYB63 and MYB85 (h) [21,60]. SND2 activates transcription of GPI-anchored *FLA11/FLA12*, *CTL2 *and other components of the signal transduction pathway (i), which leads to upregulation of *SND1 *(a).

Recently, a signal transduction pathway based on a mammalian signalling model was proposed for SCW biosynthesis in *Arabidopsis *and rice (Figure 4 in [56]). Differentially expressed genes in SND2∩Ko included those encoding the principal proteins of this machinery (Table 1), namely *FLA11/FLA12*, *CTL2 *(AT3G16920), an LRR kinase (AT1G08340), Rac (AT2G36570), IQ (*IQD10*, AT3G15050) and RIC (AT1G27380). *CHITINASE-LIKE 2 *(*CTL2*), which lacks chitinase or chitin-binding activity [66], might interact with *FLA11/12 *in a similar way to the interaction of mammalian chitinase-like protein SI-CLP with a fasciclin domain-containing transmembrane receptor, Stabilin-1 [56,67]. Two additional kinases (AT1G09440 and AT1G56720, Table 1) could possibly be involved in this signalling cascade. Based on these findings, we propose a revised model for the role of SND2 in the transcriptional network underlying fibre SCW deposition (Figure 5). Under this model, SND2 directly or indirectly upregulates the genes associated with this signalling machinery. The nature of this regulatory relationship remains to be resolved.

Despite the upregulation of the associated biosynthetic genes, we did not observe corresponding relative increases in glucose (i.e. cellulose) or xylose (i.e. xylan) content per unit mass (Table 4). There may not be a direct relationship between *CesA *expression and cellulose content, as evidenced when *SND1 *is overexpressed [28]. However, we found that mannose and rhamnose content of stems were significantly increased in SND2-OV(A) by 7.4% and 5.4%, respectively (Table 4). The increase in mannose could be explained by the upregulation of *CslA9 *(Table 1), since CSLA proteins encode β-mannan synthases [68,69]. Rhamnose and mannose were also reported to be increased due to *SND1 *overexpression [28].

Although we found no effect on fibre SCW thickness in homozygous SND2-OV lines A and B, the fibre SCW thickness of line C was significantly and reproducibly decreased relative to wild type (Figure 3; Additional file 1, Figure S4). Because line C exhibited the highest *SND2 *transcript abundance amongst the homozygous lines (Additional file 1, Figure S1), we confirmed using several T1 *SND2*-overexpressing lines, with *SND2 *transcript far exceeding that of SND2-OV(A), that strong *SND2 *overexpression reduces fibre SCW thickness (Figure 4). This phenotype resembles the dominant repression phenotype of *SND2*, rather than the overexpression phenotype, reported previously [21]. However, due to the stable expression of *SND2 *transcript in all transgenic lines (Additional file 1, Figure S1; Figure 4), this cannot be explained by co-suppression. Interestingly, a similar paradox has been observed for *SND1 *overexpression [20,28], where excess levels of this transcriptional activator were reported to have an indirect repressive effect. We suggest that this phenomenon could be attributed to gene dosage effects, where a stoichiometric increase in one TF protein leads to a decreased molar yield of a multi-protein complex, and greater yield of incomplete intermediates (reviewed by Birchler *et al. *[70]). Such a phenomenon could also explain the observation that *CesA8 *upregulation was restricted to the most moderate *SND2*-overexpressing line, SND2-OV(A). Notably, the transgenic lines in our study expressed *SND2 *at least an order of magnitude greater than the ~16-fold expression levels reported for lines with increased fibre wall thickness by Zhong *et al. *[21]. This is likely due to a double, rather than a single, *CaMV 35S *promoter in the pMDC32 vector driving *SND2 *overexpression in this study. Because we failed to identify SND2-OV lines with *SND2 *abundance near the range of 16-fold, we cannot preclude that limited *SND2 *overexpression may increase fibre SCW thickness.

Interestingly, when we overexpressed *Arabidopsis SND2 *in *Eucalyptus *xylem (Table 5), we observed a phenotype in better agreement with that previously reported for *Arabidopsis *[21]. *SND2 *overexpression in *Eucalyptus *significantly increased fibre cell area, likely due to increased cell wall area (Table 5). Because our assessment of *SND2 *overexpression in multiple independent events in both *Arabidopsis *and *Eucalyptus *contrast not only with each other but also with that of Zhong *et al. *[21], our results suggest that the phenotypic effects of *SND2 *gain-of-function mutagenesis are intrinsically variable. The positive effect of *SND2 *overexpression on *Eucalyptus *fibre development could be the result of a greater tolerance in *Eucalyptus *to high-abundance SND2 and/or SND2 co-regulator levels in woody xylem, since more carbon is allocated to SCW biosynthesis in *Eucalyptus *than in *Arabidopsis*. Alternatively, *SND2 *transcript levels remained moderate in *Eucalyptus*, a possibility that cannot be explored using the Induced Somatic Sector Analysis technique.

In addition to the requirement of the appropriate level of SND2 abundance in *Arabidopsis*, spatial and temporal expression of a co-regulator(s) is a further requirement. The fibre-restricted SCW phenotype of *SND2 *overexpression observed in *Arabidopsis *by Zhong *et al. *[21] illustrates the requirement of a spatially regulated co-regulator(s) for SND2 to activate its targets, which is presumably also expressed in fibres. Our results support this observation. Due to the fact that none of the genes differentially expressed at eight weeks (Table 1) were differentially expressed in four week stems (Additional file 4), we further suggest that the co-regulator(s) is temporally regulated, and that the temporal regulation of the co-regulator(s) may be a limiting factor that constrains the ability of SND2 to activate its native target genes at four weeks.

## Conclusions

Our results suggest that *SND2 *regulates genes involved in cellulose, mannan, and xylan biosynthesis, cell wall modification, and lignin polymerization, but not monolignol biosynthesis. SND2 also promotes upregulation of a relatively small number of TFs, amongst them *MYB103 *and *SND1*. We implicate *SND2 *in the unexpected regulation of the machinery of a signal transduction pathway proposed for SCW development [56] and propose a model in which SND2 occupies a subordinate but central position in the transcriptional regulatory network (Figure 5), with possible indirect positive feedback to higher regulators and signalling pathways. Our data support the role of *SND2 *in fibre SCW transcriptional regulation, but our study suggests that, at excessive levels of overexpression, *SND2 *has a negative effect on IF SCW deposition. This phenomenon requires further investigation. We postulate that *SND2 *overexpression could increase SCW deposition within a limited range of overexpression, relying in part on the abundance of additional regulator proteins. However, we show that *SND2 *overexpression has the potential to enhance fibre development in *Eucalyptus *trees, an important commercial forestry crop.

## Methods

### Plant growth conditions

*Arabidopsis thaliana *Columbia (Col-0) plants were grown in peat moss bags (Jiffy Products International AS, Norway) under a 16 h day artificial light regime, at ~22°C and ~75% humidity with weekly fertilization. Where applicable, hygromycin selection was performed for ~14 days before transferral of seedlings to peat moss bags. The stated age of the plants is inclusive of the hygromycin selection period.

### Generation of overexpression constructs and transformation

The coding sequence of *SND2 *(AT4G28500) was amplified (forward primer, 5'-ATGACTTGGTGCAATGACCGTAG-3', reverse primer 5'-TTAAGGGATAAAAGGTTGAGAGTCAT-3') from *Arabidopsis thaliana *Col-0 inflorescence stem cDNA. The amplicon was gel-purified with the MinElute Gel Extraction Kit (Qiagen, Valencia, CA) and cloned into pCR8/GW/TOPO as per the manufacturer's instructions (Invitrogen, Carlsbad, CA). The sequenced insert was transferred to pMDC32 and pCAMBIA1305.1 [35] using the Gateway LR Clonase™ II Enzyme Mix (Invitrogen). The construct was introduced into *Agrobacterium tumefaciens *strains LBA4404 and AGL1 for pMDC32 and pCAMBIA1305.1 constructs, respectively, followed by *Agrobacterium-*mediated transformation of *Arabidopsis thaliana *Col-0. After surface sterilization, transgenic seed was selected on 0.8% agar containing 20 μg/ml Hygromycin B. The seeds were artificially stratified at 4°C for 2-4 days prior to germination at 22°C under artificial illumination.

### Microarray analysis

For the eight-week experiment, T4 seedlings were selected on hygromycin for two weeks and grown in peat moss bags for six weeks. For the four week experiment, no selection was employed; homozygous T4 seeds were germinated directly on peat moss. Each of three biological replicates consisted of ten or six plants in the four and eight week experiments, respectively. Stem tissues were collected on the same day between 08:30 and 11:00, flash-frozen in liquid nitrogen and stored at -80°C. Total RNA extracted from the bottom 100 mm of the primary inflorescence stems was treated with the RNase-free DNase Set (Qiagen) and genomic DNA contamination assessed by PCR using intron-spanning primers. RNA integrity was quantified using the Experion™ instrument (Bio-Rad Laboratories, Inc.). cDNA synthesis and cyanine dye coupling were performed as prescribed by the African Centre for Gene Technologies (ACGT) Microarray Facility (available at http://www.microarray.up.ac.za/MA008_indirect_labelling_version3.pdf).

Microarray hybridization was performed using the *Arabidopsis thaliana *4 × 44k DNA microarray V3 (Agilent Technologies, Santa Clara, CA), as described by the manufacturer's instructions, but substituting cRNA with cDNA. Dye-swaps were employed to correct for fluorophore bias. Slides were scanned using an Axon GenePix 4000B instrument (Axon Instruments, Foster City, CA, USA). Features were extracted using Axon GenePix Pro software (v6.0) and imported into limma (linear models for microarray data) [71]. Data were normalized in R as described by Crampton *et al. *[72], with linear models based on the comparison between SND2-OV(A) and the wild type, analyzing each time point independently. Significant DEGs were defined as those with P_c_-value < 0.05, where P_c _is the False Discovery Rate. Raw data files of all the microarray experiments are available from the Gene Expression Omnibus http://www.ncbi.nlm.nih.gov/projects/geo/, under accession number GSE29693.

Differentially expressed genes were subjected to an anatomical meta-analysis of expression in selected *Arabidopsis *tissues by hierarchical clustering (Pearson correlation) in the Genevestigator V3 public microarray database [39]. Only high quality ATH1 22k arrays, and probe sets highly specific for a single gene, were selected for analysis.

### Reverse Transcription Quantitative Polymerase Chain Reaction (RT-qPCR) analysis

The quality of total RNA extracted from lower inflorescence stems was assessed by Experion™ analysis (Bio-Rad Laboratories, Hercules, CA). First-strand cDNA synthesis from genomic DNA-free RNA was performed using the Improm-II™ Reverse Transcriptase cDNA synthesis kit (Promega, Madison, WI) and cDNA purified using the RNeasy Mini Kit (Qiagen). RT-qPCR reactions were quantified using the LightCycler 480 system [45 cycles of 95°C denaturation (10s), 60°C annealing (10s) and 72°C extension (15s)] (Roche GmbH, Basel, Switzerland). Primer sequences that were used for each gene target are listed in Additional file 3, Table S2. LightCycler 480 Software v. 1.5.0. (Roche) was used for second derivative maximum value calculation and melting curve analysis. Statistical analysis was performed with Biogazelle qBasePLUS [73].

### Microscopy

For light microscopy, the lower ~5 mm of the primary inflorescence stem was fixed in formaldehyde/glutaraldehyde buffer (3.5% and 0.5% v/v, respectively) for up to five days and dehydrated in an ethanol series before embedding in LR White™ resin. Stem sections of 0.5 μm thickness were visualized with Toluidine Blue. Micrograph measurements were performed using ImageJ software (National Institutes of Health, http://rsbweb.nih.gov/ij/), using the polygon tool for cell area measurements. For SEM, 90 nm thick epoxy-embedded samples were imaged following sodium methoxide etching for 1 min [74] using a LEO 1455 VP-SEM instrument (Carl Zeiss, Germany) at 5 kV.

### Klason Lignin and Cell Wall Sugar Analysis

Complete inflorescence stems from eight-week-old transgenic and wild type plants were stripped of siliques and cauline leaves and dried (100°C, 24 h). Stems from up to 24 plants were pooled for each of three biological replicates. Cell wall sugar and Klason lignin analysis were performed essentially as described by Coleman *et al *[75], using High Performance Liquid Chromatography (Dionex CarboPac PA1 4 × 250 mm) to determine carbohydrate concentrations. Triplicate technical repetitions were performed.

### Induced Somatic Sector Analysis (ISSA)

ISSA was performed as described before [42] with some modifications. Eleven ramets of each of two hybrid clones, *E. grandis *x *E. camaldulensis *and *E. camaldulensis *x *E. globulus*, were selected in early summer on the basis of good form and growth for experimentation and ten 1 cm^2 ^cambial windows were created on each plant. *Agrobacterium tumefaciens *AGL1 harbouring pCAMBIA1305.1 containing the *Arabidopsis SND2 *CDS and the *β*-glucuronidase or 'GUS' reporter gene was injected into the cambial windows. Plants were fertilised after inoculation and maintained in the glasshouse in the same condition as described previously [42] until harvest. After 195-210 days cambial windows were excised from the main stem, the phloem portion was removed and the remaining xylem tissue was washed twice with 0.1 M NaPO_4 _buffer (pH 7). Transgenic sectors were identified by GUS reporter staining. Eleven *SND2*-overexpressing and nine empty vector control sectors were analyzed. Transgenic sectors were excised in blocks of 1-3 mm^3 ^(from the cambium to wound parenchyma) using a single edge razor blade, so that the sector was located close to the middle of the block when viewed on the longitudinal tangential plane. Blocks were then sliced transversely through the middle of the sector to expose the transverse surface of the sector, and then mounted with conductive adhesive on SEM stubs. Transgenic sectors were delineated within the block by etching the borders of the GUS reporter stain with a razor blade. Blocks were desiccated overnight prior to SEM imaging. Cell morphology measurements were undertaken using the Quanta Environmental Scanning Electron Microscope (FEI, Hillsboro, Oregon) to investigate changes in cell wall thickness, cell wall area (total amount of cell wall), cell area and lumen area. Images were taken of both transgenic sector and directly adjacent non-transgenic tissue, twenty to fifty cells from the cambial surface, using the low vacuum mode. Images were then analysed using freeware Image-J http://rsbweb.nih.gov/ij/ with ten fibres measured per micrograph. For the cell wall thickness, the mean of three measurements for each cell wall were used for cell wall thickness calculations, whilst for the remaining properties one value for each fibre was sufficient. Average values were calculated for each sector and their non-transgenic control tissues and converted into percentage change values. Percentage change values between *SND2 *overexpression sectors and empty vector control (EVC) were statistically assessed with the Student's t-test.

## List of abbreviations used

DEG: differentially expressed gene(s); EVC: empty vector control; FDR: false discovery rate; GO: gene ontology; IF: interfascicular fibre; ISSA: Induced Somatic Sector Analysis; NST: NAC SECONDARY WALL THICKENING PROMOTING FACTOR; SCW: secondary cell wall; SEM: scanning electron microscopy; SND: SECONDARY WALL-ASSOCIATED NAC DOMAIN; SWN: secondary wall NAC: TF: transcription factor; VND: VASCULAR-RELATED NAC DOMAIN.

## Authors' contributions

SGH conducted the experimental work and drafted the manuscript. EM, DKB and AAM assisted with the drafting of the manuscript, conceived of the project and aided in the design and supervision of the study. AVS and GB performed the induced somatic sector analysis experiments. All authors have reviewed and approved the manuscript.

## Supplementary Material

Additional file 1 **Figure S1**. Figure S2. Figure S3. Figure S4. Figure S5. Figure S6. Figure S7. Click here for file

Additional file 2 **Microarray data of SND2-OV(A) vs. wild type (8 weeks), fold change > |±1.5|**. List of significantly differentially expressed genes of SND2-OV line A stems at 8 weeks, compared to the wild type, with fold change values larger than 1.5. Click here for file

Additional file 3 **Additional file 3, Table S1**. Additional file 3, Table S2. Click here for file

Additional file 4 **Microarray data SND2-OV(A) vs. wild type (4 weeks)**. List of significantly differentially expressed genes of SND2-OV line A stems at 4 weeks, compared to the wild type. Click here for file
