# Supplementary material for: SND2, a NAC transcription factor gene, regulates genes involved in secondary cell wall development in Arabidopsis fibres and increases fibre cell area in Eucalyptus
Source: BMC Plant Biol. 2011 Dec 1;11:173. doi: 10.1186/1471-2229-11-173 (PMC3289092; doi:10.1186/1471-2229-11-173)
Supplement: Additional file 1 — Figure S1. Figure S2. Figure S3. Figure S4. Figure S5. Figure S6. Figure S7. [file 1471-2229-11-173-S1.PDF]

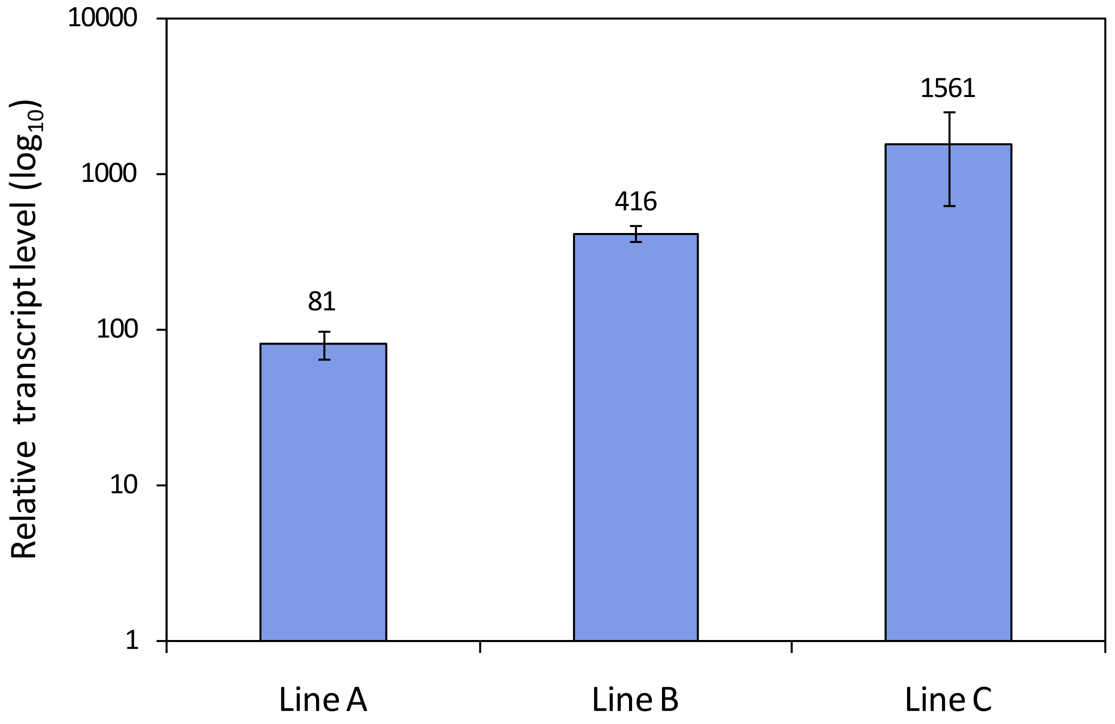

Figure S1. RT-qPCR analysis of total *SND2* transcript in lower inflorescence stems of T4 SND2-OV lines A, B and C, relative to the wild type. The primer pair quantifies both endogenous and transgenic *SND2* transcript. Calibrated Normalized Relative Quantity (CNRQ) values were obtained by normalizing against three control genes (*ACTIN2*, *UBIQUITIN5*, *EF1α*). SND2-OV transcript levels were normalized to the wild type (assigned a value of 1) in each replicate, hence error bars indicate the standard error of the deviation from wild type across biological replicates. The means are indicated above each bar.

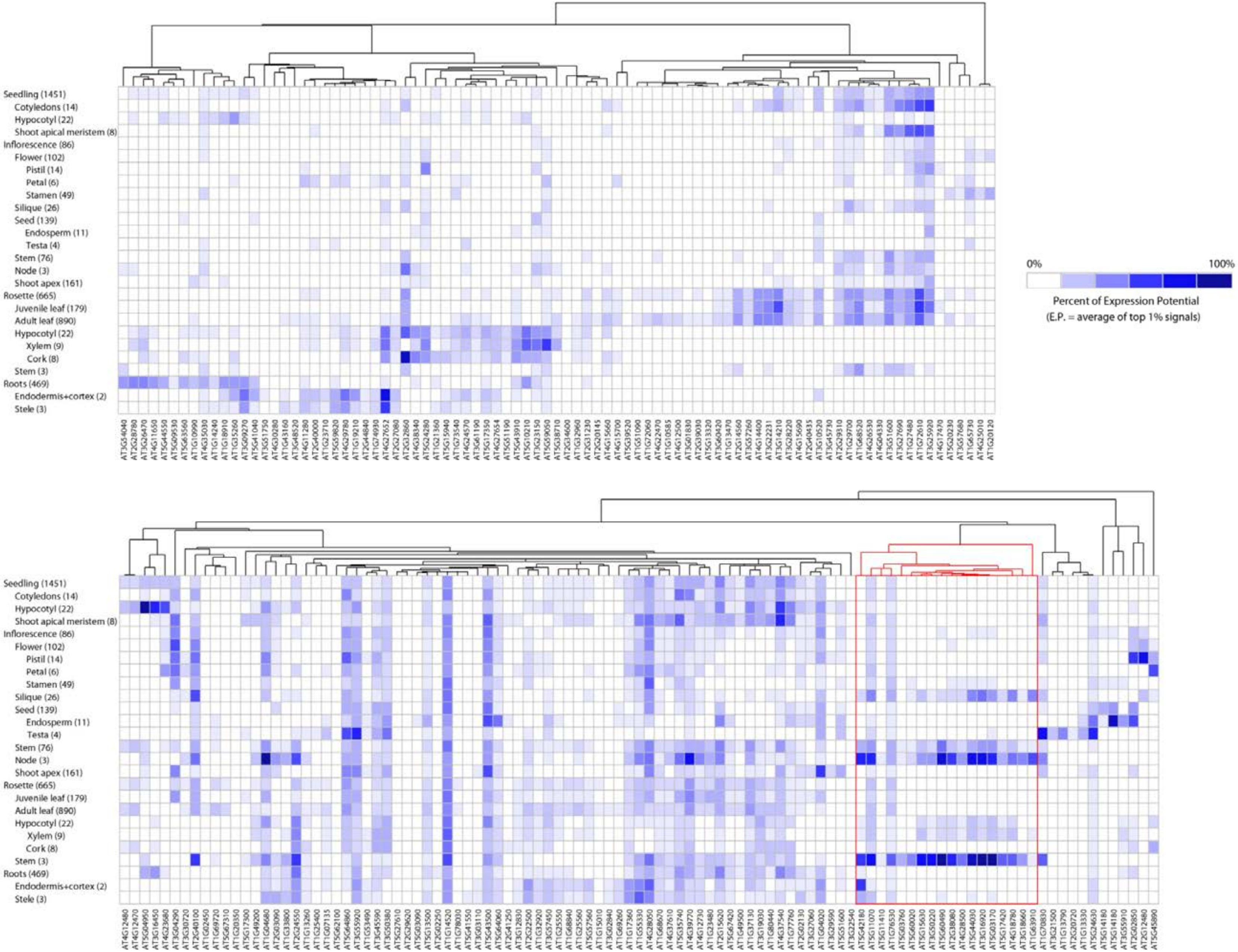
Seedling (1451)
Cotyledons (14)
Hypocotyl (22)
Shoot apical meristem (8)
Inflorescence (86)
Flower (102)
Pistil (14)
Petal (6)
Stamen (49)
Silique (26)
Seed (139)
Endosperm (11)
Testa (4)
Stem (76)
Node (3)
Shoot apex (161)
Rosette (665)
Juvenile leaf (179)
Adult leaf (890)
Hypocotyl (22)
Xylem (9)
Cork (8)
Stem (3)
Roots (469)
Endodermis+cortex (2)
Stele (3)
0%
100%
Percent of Expression Potential
(E.P. = average of top 1% signals)

Previous page

Figure S2. Heat map of absolute transcript abundance of genes represented on ATH1 22k arrays, that were differentially expressed by at least 1.5-fold in SND2-OV(A) stems relative to wild type, in various *Arabidopsis* tissues and organs. Genevestigator V3 [1] was used for microarray data mining, and the anatomical cluster analysis tool was used to visualize and cluster the genes according to their tissue-specific expression patterns. Tissues/organs are staggered hierarchically, and the number of arrays on which the data are based is indicated in parenthesis. Absolute transcript values are expressed as a percentage of their expression potential (E.P.), where E.P. is the mean of the top 1% of hybridization signals for a given probe set across all arrays. The cluster highlighted in red is comprised of 18 genes, including *SND2* (AT4G28500), which displayed preferential expression in tissues and organs containing secondary cell walls.

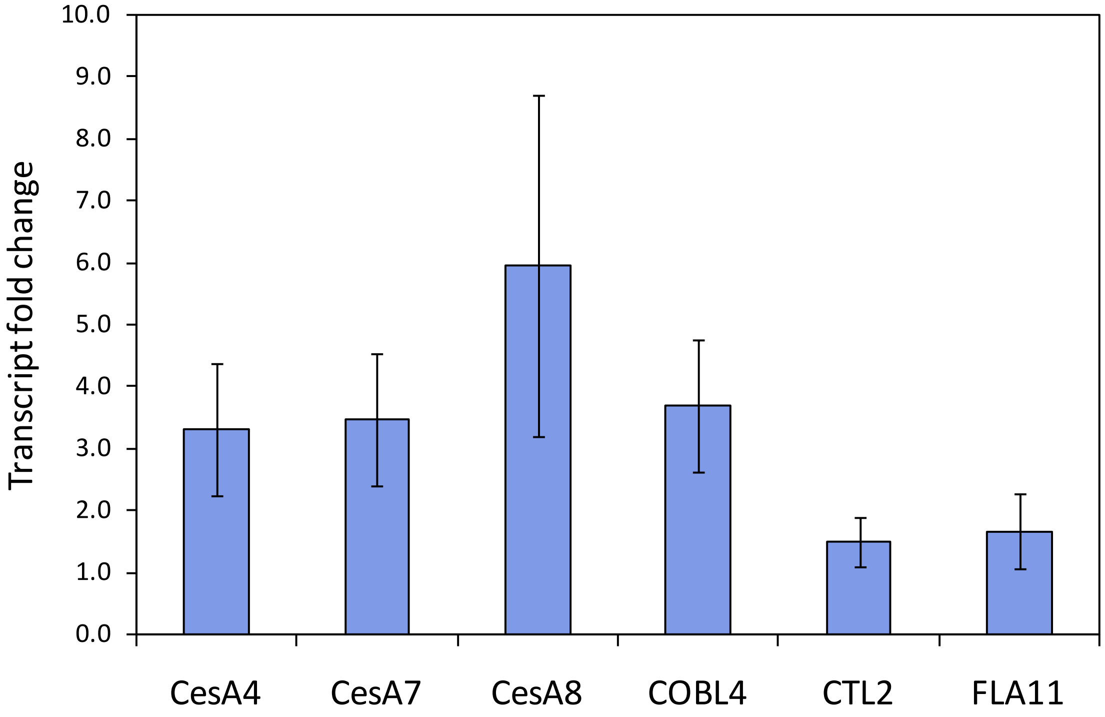

Figure S3. Fold change of RT-qPCR analysis of selected genes differentially expressed in SND2-OV(A) inflorescence stems at eight weeks, from an independent trial to that of the microarray analysis. SND2-OV(A) plants were grown alongside the wild type in three paired biological replicates, where primary inflorescence stems from ten plants were pooled per replicate. SND2-OV(A) transcript levels were normalized to the wild type in each replicate (assigned a value of 1, for each gene), hence error bars indicate the standard error of the deviation from wild type across biological replicates.

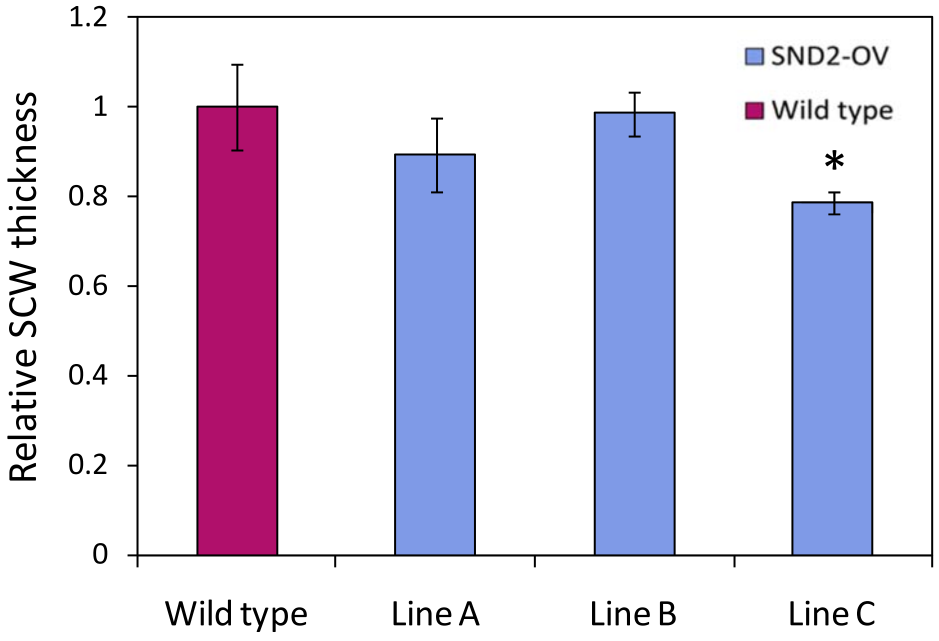

Figure S4. Relative secondary cell (SCW) wall thickness in interfascicular fibres of eight-week-old wild type and T4 SND2-OV lines, determined by light microscopy. Measurements were obtained from Toluidine Blue-stained 400X images and are normalized to wild type. Error bars indicate the standard error of the mean of three plants, where for each plant between two and five interfascicular fibre regions were measured and an average value obtained across regions (10 to 66 fibres measured per plant). Samples were obtained from an independent trial to that of the SEM analysis. *Significantly different from the wild type based on a two-tailed Student's t-test ($P < 0.05$).

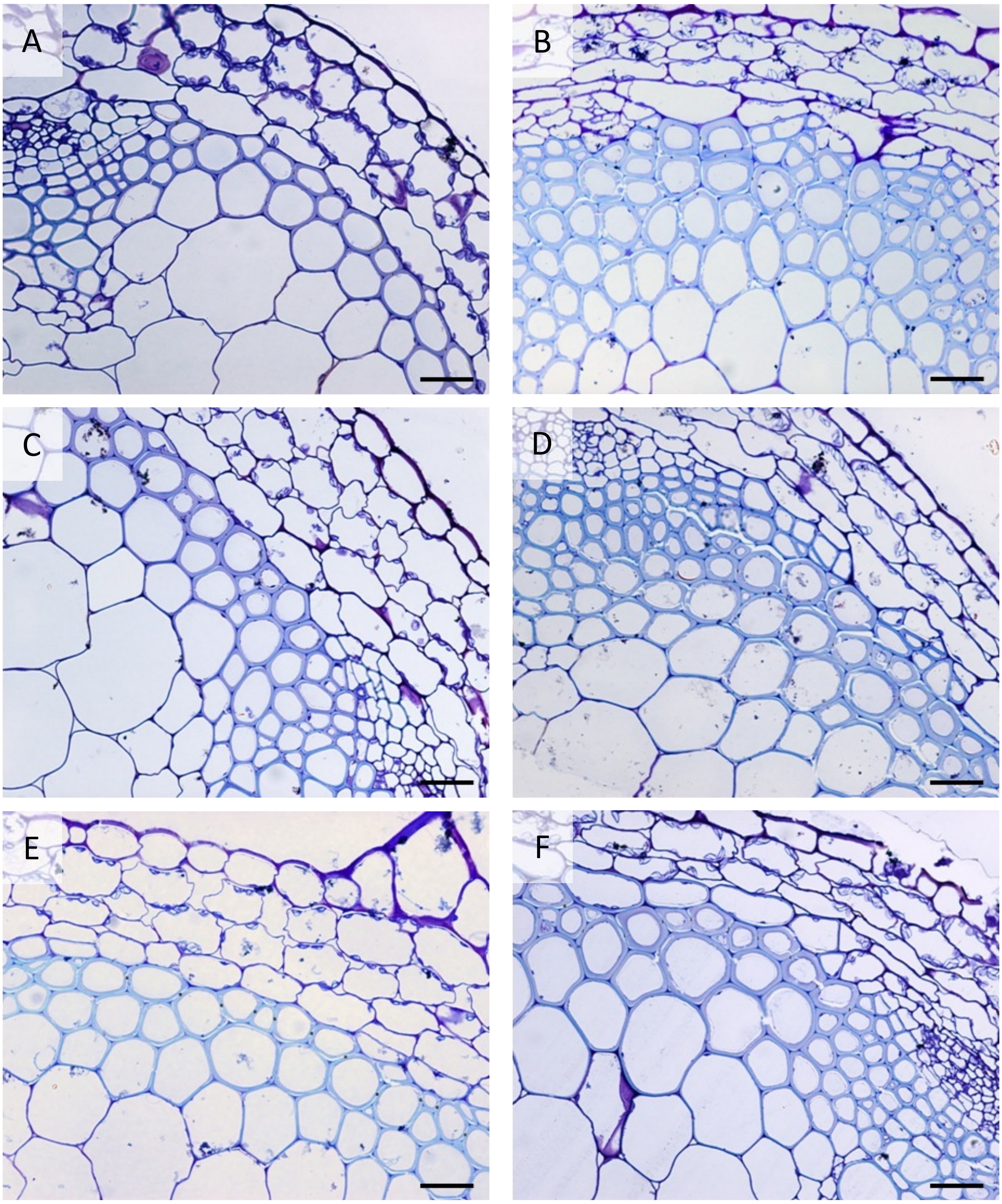

Figure S5. Light microscopy images showing decreased interfascicular fibre SCW thicknesses in T1 SND2-OV plants. Sections were stained with toluidine blue. *A*, *C*, *E*, T1 SND2-OV inflorescence stem cross-sections; *B*, *D*, *F*, wild type. Scale bars = 20 µm.

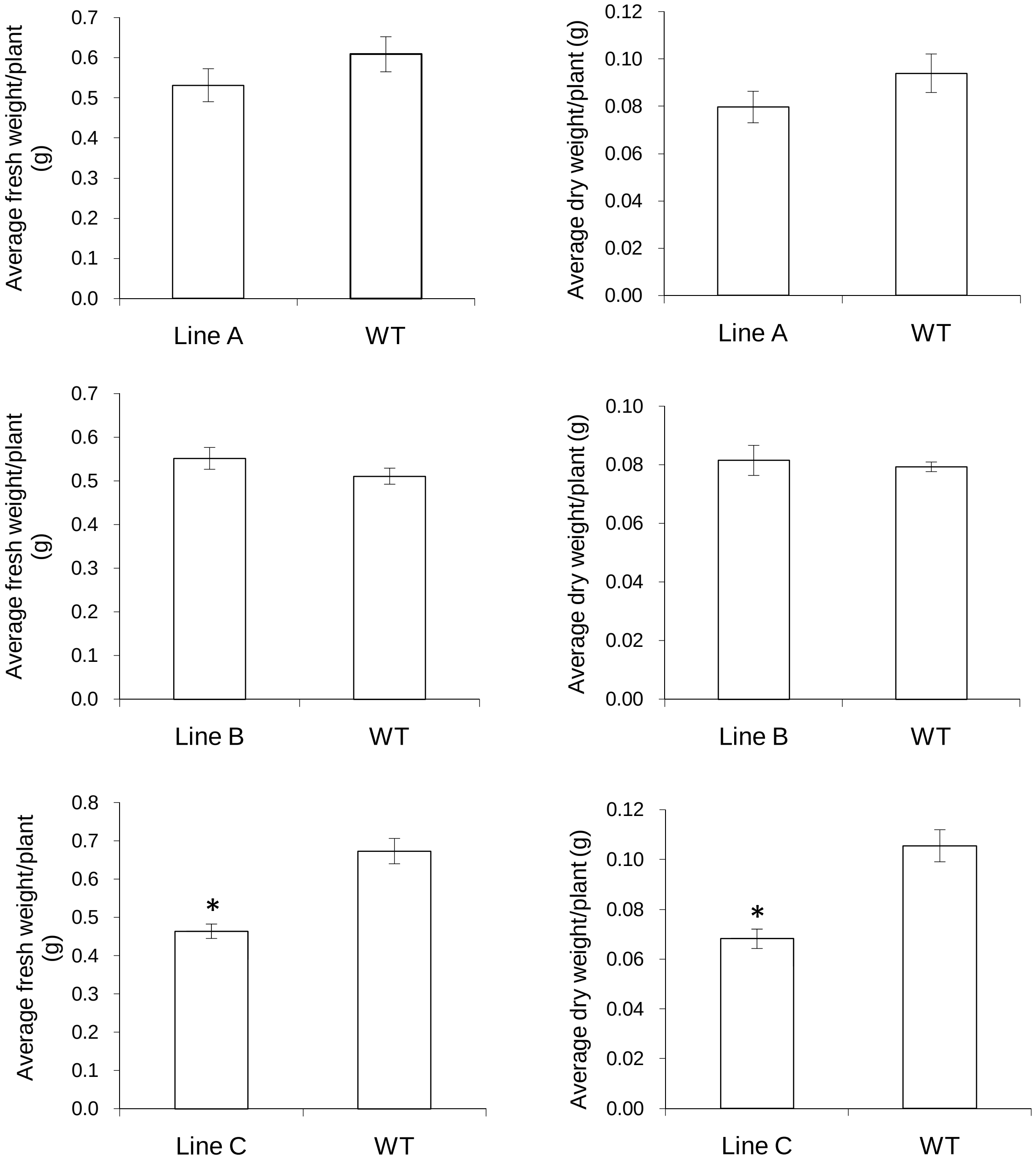

Figure S6. Fresh and dry inflorescence stem biomass of eight-week-old wild type (WT) and T4 SND2-OV lines A, B, and C. Error bars indicate standard error of the mean of three biological replicates (stems from approximately nine plants were pooled per replicate). *Significantly different from the wild type according to homoscedastic two-tailed Student's t-test ($P < 0.001$). For lines A and C, similar results were obtained in an independent trial (data not shown).

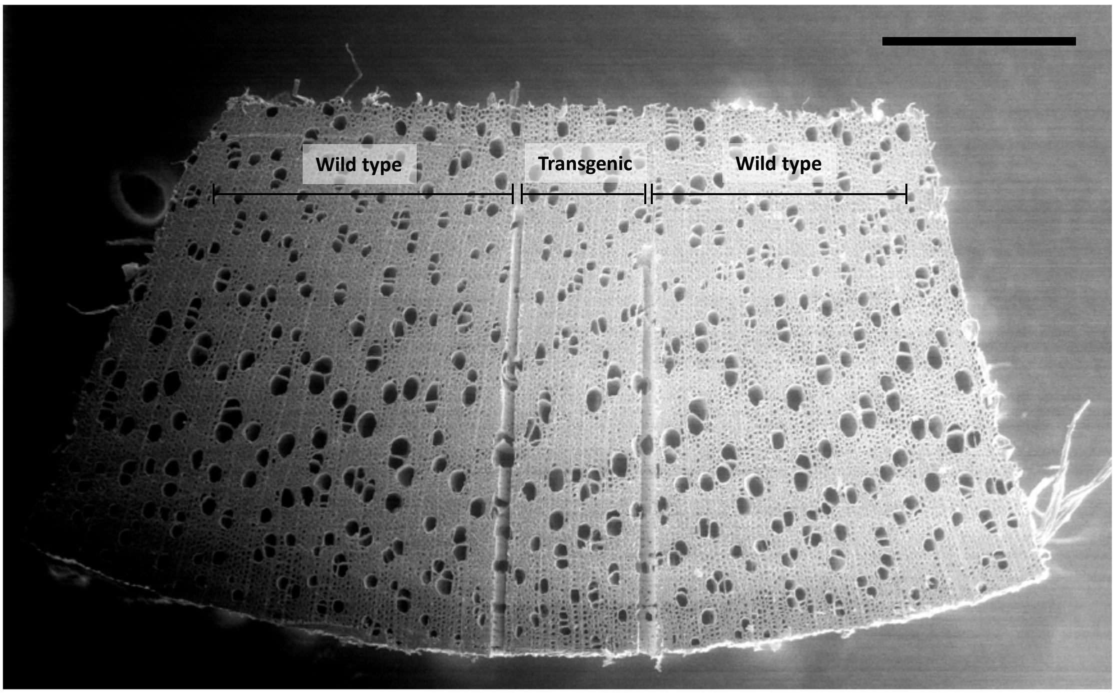

Figure S7. Scanning electron micrograph of a *Eucalyptus* induced somatic sector. The transgenic sector has been marked by etching the sample either side of the GUS reporter stain (not visible). Scale bar = 500 µm.

References

1. Hruz T, Laule O, Szabo G, Wessendorp F, Bleuler S, Oertle L, Widmayer P, Gruissem W, Zimmermann P: **Genevestigator V3: A reference expression database for the meta-analysis of transcriptomes**. *Adv Bioinformatics* 2008, **2008**:420747.
