# Supplementary material for: SND2, a NAC transcription factor gene, regulates genes involved in secondary cell wall development in Arabidopsis fibres and increases fibre cell area in Eucalyptus
Source: BMC Plant Biol. 2011 Dec 1;11:173. doi: 10.1186/1471-2229-11-173 (PMC3289092; doi:10.1186/1471-2229-11-173)
Supplement: Additional file 3 — , Table S1. Additional file 3, Table S2. [file 1471-2229-11-173-S3.PDF]

Table S1. Enriched biological processes associated with genes differentially expressed in stems of eight-week-old SND2-OV(A) plants, relative to wild type, according to the GoToolBox resource [1]. Terms exclusive to category levels higher than 6 or below level 4 were excluded for simplicity. The GO identification number and level of each biological process is also indicated.

GO ID	Level	GO Term	P-value[a]	Enrichment[b]
**Cell wall organization or biogenesis (GO:0071554)**				
GO:0009834	5	Secondary cell wall biogenesis	3.25E-08	52.33
GO:0009832	4	Plant-type cell wall biogenesis	6.23E-06	19.63
GO:0009664	5	Plant-type cell wall organization	4.52E-03	9
GO:0010382	5	Cellular cell wall macromolecule metabolic process	8.94E-03	63
**Carbohydrate metabolic process (GO:0005975)**				
GO:0044042	6	Glucan metabolic process	1.51E-05	11.42
GO:0044264	5,6	Cellular polysaccharide metabolic process	5.91E-05	8.77
GO:0005976	5	Polysaccharide metabolic process	9.55E-05	8.02
GO:0044262	4,5	Cellular carbohydrate metabolic process	3.93E-04	3.96
GO:0000271	5,6	Polysaccharide biosynthetic process	2.17E-03	7.2
GO:0034637	5,6	Cellular carbohydrate biosynthetic process	2.60E-03	5.15
GO:0016051	4,5	Carbohydrate biosynthetic process	7.81E-03	3.88
**Signalling process (GO:0023046)**				
GO:0000160	6,5	Two-component signal transduction system (phosphorelay)	3.54E-05	13.65
GO:0007242	6,5	Intracellular signalling cascade	7.05E-03	2.7
**Response to stimulus (GO:0050896)**				
GO:0051707	3,4	Response to other organism	1.84E-10	6.78
GO:0009620	4,5	Response to fungus	7.08E-08	12.04
GO:0009611	4	Response to wounding	3.76E-07	11.98
GO:0006952	4	Defence response	1.99E-06	4.04
GO:0009617	4,5	Response to bacterium	3.46E-06	7.45
GO:0009725	4	Response to hormone stimulus	4.22E-06	4.03
GO:0009723	5	Response to ethylene stimulus	5.34E-06	10.23
GO:0009751	4	Response to salicylic acid stimulus	5.81E-06	10.23
GO:0050832	5,6	Defence response to fungus	8.69E-06	12.57
GO:0009816	6,5,7	Defence response to bacterium, incompatible interaction	1.59E-05	25.2
GO:0010200	6	Response to chitin	2.28E-05	10.47
GO:0042742	5,6	Defence response to bacterium	2.48E-05	8.15
GO:0009814	5,4,6	Defence response, incompatible interaction	3.20E-05	9.92
GO:0009743	5	Response to carbohydrate stimulus	2.71E-04	6.61
GO:0010033	4	Response to organic substance	3.01E-04	6.5
GO:0009409	5,4	Response to cold	7.00E-04	5.54
GO:0009753	4	Response to jasmonic acid stimulus	7.34E-04	6.98
GO:0032870	4,5	Cellular response to hormone stimulus	9.63E-04	5.16
GO:0009755	7,6,5	Hormone-mediated signalling	9.63E-04	5.16
GO:0009733	5	Response to auxin stimulus	2.09E-03	4.38
GO:0009612	4	Response to mechanical stimulus	2.26E-03	25.2
GO:0045087	4,5	Innate immune response	3.58E-03	3.93
GO:0016046	5,6	Detection of fungus	4.49E-03	126
GO:0009266	4	Response to temperature stimulus	5.10E-03	3.63
GO:0009416	5	Response to light stimulus	5.33E-03	3.14
GO:0009314	4	Response to radiation	6.16E-03	3.06
GO:0006979	4	Response to oxidative stress	6.37E-03	4.08
**Nitrogen compound metabolic process (GO:0006807)**				
GO:0046209	4	Nitric oxide metabolic process	1.19E-04	126
GO:0006809	6,5	Nitric oxide biosynthetic process	1.19E-04	126
GO:0042128	5	Nitrate assimilation	8.70E-04	42
GO:0042126	4	Nitrate metabolic process	1.06E-03	42
**Transport (GO:0006810)**				
GO:0006869	4,5	Lipid transport	2.01E-03	5.41

Table S1. *(continued from previous page)*

GO ID	Level	GO Term	P-value[a]	Enrichment[b]
**Interspecies interaction between organisms (GO:0044419)**				
GO:0052095	4,6,5	Formation of specialized structure for nutrient acquisition from other organism during symbiotic interaction	4.49E-03	126
GO:0044002	5,6	Acquisition of nutrients from host	4.49E-03	126
GO:0051816	4,5	Acquisition of nutrients from other organism during symbiotic interaction	4.49E-03	126
**Biological regulation (GO:0065007)**				
GO:0009889	5,4	Regulation of biosynthetic process	6.52E-03	1.82
GO:0031326	6,5	Regulation of cellular biosynthetic process	6.52E-03	1.82
GO:0050794	4,3	Regulation of cellular process	7.83E-03	1.55
GO:0080090	5,4	Regulation of primary metabolic process	8.30E-03	1.77
GO:0030307	6,7,4,5	Positive regulation of cell growth	8.94E-03	63

[a]Benjamini & Hochberg correction [2]

[b]Fold enrichment of each GO term is defined as the proportion of genes in the microarray dataset annotated by a GO term, relative to the genome-wide annotation of the GO term.

Table S2. Primer sequences used for RT-qPCR analysis.

Gene target	TAIR Locus	Primer sequences (5'→3')
**Target genes**		
SND2 (endogenous)	AT4G28500	Forward: TGATGAAGTTGTGAGCACTGAA
		Reverse: TGACAAGAGACCGGAAGTGA
SND2 (total)	N/A	Forward: TCACTTCCGGTCTCTTGTCA
		Reverse: TGCGTCATCTCTTACCTTGC
MYB103	AT1G63910	Forward: GTCGTCATCAACCGTCAGTA
		Reverse: TCGATGTTGTGGTGGTAGAG
COBL4	AT5G15630	Forward: TAGAGTCCACTGGCACGTTA
		Reverse: CCTGAAGGTCCAGCTTCCAT
CesA4	AT5G44030	Forward: TTGGTGTTGTTGCCGGAGTT
		Reverse: AACAGTCGACGCCACATTGC
CesA7	AT5G17420	Forward: CGTTGTTGCAGGCATCTCAG
		Reverse: AGCAGTTGATGCCACACTTG
CesA8	AT4G18780	Forward: CCGCAATCTTCATCATCGTC
		Reverse: CCGCCATTCTCCATAAGAGT
CslA09	AT5G03760	Forward: ACACCAAGGTCATTGCATCT
		Reverse: TACACCGAGTTCCAACACAT
EXPA15	AT2G03090	Forward: GTCCTCCTAACAACGCTCTT
		Reverse: CGCAACCGAATGAACATCTC
FLA12	AT5G60490	Forward: ATGTCTACAGCGATGGACAG
		Reverse: CCATGCGAGCATTACACTCA
AGP21	AT1G55330	Forward: ATGGAGGCAATGAAGATGAAG
		Reverse: AACATGGCAGCATCAGAAGTT
AT1G20120	AT1G20120	Forward: ACCAGTTGTACCGGCATATT
		Reverse: TTGACATCGGTATCGCACTT
AT5G11410	AT5G11410	Forward: CACGGGTCAAGATAGCCATA
		Reverse: GTGTTGTAGTGCTCGTCAAG
VSP1	AT5G24780	Forward: AGTCCGGAGAATCAACTCCA
		Reverse: GTACACCACTTGCGTCAACT
AT3G01345	AT3G01345	Forward: AATGGACGCCTTGCTATCAG
		Reverse: AGGCTTCGGTAACACCTACT
FLA11	AT5G03170	Forward: GTGGCGATGATGGAGGAGAT
		Reverse: CAATGGCTGCAACGGTAGTG
CTL2	AT3G16920	Forward: CTGCAACAGCGGATTCGATA
		Reverse: AGTCACCGAACCAGAGGTTA
**Control genes**		
ACT2	AT3G18780	Forward: TGGAATCCACGAGACAACCT
		Reverse: TGGACCTGCCTCATCATACT
EF1α	AT1G07920	Forward: ACAGGCGTTCTGGTAAGGAG
		Reverse: CCTTCTTGACGGCAGCCTTG
UBQ5	AT3G62250	Forward: GGTGGTGCTAAGAAGAGGAA
		Reverse: TCGATCTACCGCTACAACAG

References

1. Martin D, Brun C, Remy E, Mouren P, Thieffr D, Jacq B: **GOToolBox: functional analysis of gene datasets based on Gene Ontology**. *Genome Biology* 2004, **5**:R101.

2. Benjamini Y, Hochberg Y: **Controlling the false discovery rate: a practical and powerful approach to multiple testing**. *Journal of the Royal Statistical Society Series B (Methodological)* 1995, **57**:289.
